# No Association of *IFNG+874T/A* SNP and *NOS2A-954G/C* SNP Variants with Nitric Oxide Radical Serum Levels or Susceptibility to Tuberculosis in a Brazilian Population Subset

**DOI:** 10.1155/2013/901740

**Published:** 2013-08-18

**Authors:** Ana Cristina C. S. Leandro, Márcia Andrade Rocha, Andreia Lamoglia-Souza, John L. VandeBerg, Valeria Cavalcanti Rolla, Maria da Gloria Bonecini-Almeida

**Affiliations:** ^1^Immunology and Immunogenetics Laboratory, Evandro Chagas Clinical Research Institute, Oswaldo Cruz Foundation, Avenida Brasil 4365, Manguinhos, 21045-900 Rio de Janeiro, RJ, Brazil; ^2^Department of Genetics and Southwest National Primate Research Center, Texas Biomedical Research Institute, 7620 NW Loop 410, 78227-5301 San Antonio, TX, USA; ^3^Tuberculosis Clinical Laboratory, Evandro Chagas Clinical Research Institute, Oswaldo Cruz Foundation, Avenida Brasil 4365, Manguinhos, 21045-900 Rio de Janeiro, RJ, Brazil

## Abstract

Tuberculosis (TB) is one of the most common infectious diseases in the world. *Mycobacterium tuberculosis* infection leads to pulmonary active disease in approximately 5–10% of exposed individuals. Both bacteria- and host-related characteristics influence latent infection and disease. Host genetic predisposition to develop TB may involve multiple genes and their polymorphisms. It was reported previously that interferon gamma (IFN-**γ**) and nitric oxide synthase 2 (NOS2) are expressed on alveolar macrophages from TB patients and are responsible for bacilli control; thus, we aimed this study at genotyping single nucleotide polymorphisms *IFNG+874T/A* SNP and *NOS2A-954G/C* SNP to estimate their role on TB susceptibility and determine whether these polymorphisms influence serum nitrite and NO_*x*_^−^ production. This case-control study enrolled 172 TB patients and 179 healthy controls. Neither polymorphism was associated with susceptibility to TB. *NOS2A-954G/C* SNP was not associated with serum levels of nitrite and NO_*x*_^−^. These results indicate that variants of *IFNG*+*874T/A* SNP and *NOS2A-954G/C* SNP do not influence TB susceptibility or the secretion of nitric oxide radicals in the study population.

## 1. Introduction 

Tuberculosis (TB) has been declared as a major global healthy threat by the World Health Organization since 1993. Pulmonary TB is highly prevalent in Brazil [1], mainly, in Rio de Janeiro, where 11,155 new cases were reported in 2011 [2]. Host factors play a major role in determining risk for active TB. Among them, IFN-*γ* production is critical in the intracellular control of *M. tuberculosis* infection, as previously demonstrated *in vitro *[3, 4] in experimental infection [5, 6]. In addition, interferon gamma (IFN-*γ*) induces apoptosis in mycobacteria-infected macrophages in a nitric oxide (NO) dependent environment [7, 8]. Polymorphism in the first intron of human *IFNG* gene is associated with higher *in vitro* production of this cytokine and is correlated with a gene dosage effect in the presence of the *IFNG+874T *allele [9]. This common polymorphism is associated with TB susceptibility in African, Turkish, Tunisian, and Central West Brazilian populations [10–13], but not in African-Americans, or people of Iranian, Hispanic, or Chinese origin [14–17].

NO is a free radical and second messenger that has been shown to be important in the development of several diseases, including TB. NO plays a major role in the pulmonary host-defense mechanism in response to infections and is implicated in bacteriostatic and bactericidal processes. NO is vital for macrophage function and granuloma formation in the immune response and kills *M. tuberculosis in vitro* [18]. NO production and NOS2 expression in rat alveolar macrophages are upregulated in response to heat-killed *M. tuberculosis* [19]. However, the role of NO in killing or limiting the growth of *M. tuberculosis* in humans is still unclear. It has been proposed that NO produced by TB-infected human macrophages and by epithelial cells exhibits antimycobacterial behavior against *M. tuberculosis* [20]. It was previously reported that the alveolar macrophages from active TB patients express inducible nitric oxide synthase (iNOS/NOS2) and may control mycobacteria growth *in vivo* [21]. Thus, the *NOS2A-954G/C *SNP may represent a pivotal protective locus against TB. Investigation of this possibility is hampered by difficulty in estimating the production of NO *in vivo *mainly in lung tissues, but genetic analysis provides a potential means of examining the relation between *NOS2A* expression and disease outcome. Given the biological and genetic validity of the role of NOS2 in the immune system, SNPs have been reported in many populations worldwide [22–24]. The *NOS2A*-954*G/C* SNP variant was originally reported in a malaria endemic area in Africa [23], suggesting that this mutation might have originated as a consequence of selective pressure of *Plasmodium* infection. In a Mexican admixed population, this functional SNP was not associated with TB [25] and no further reports have associated it with NO radical levels. 

In this present case-control study, we investigated the influence of the *IFNG+874T/A* (rs2430561) and *NOS2A-954G/C* (rs1800482) SNPs on TB susceptibility in a highly exposed and admixed population of TB patients. We also conducted functional studies to determine whether the modulation of nitric oxide radical secretion varies according to *NOS2A-954G/C * or *IFNG/NOS2A *combined genotypes.

## 2. Materials and Methods 

### 2.1. Study Population

Patients and control groups were recruited from Evandro Chagas Clinical Research Institute at Fiocruz and from Municipal Health Centers, Rio de Janeiro, Brazil. All volunteers included in this study lived in the metropolitan area of Rio de Janeiro City (RJ, Brazil), were older than 18 years, and provided written informed consent. Cases and control groups were matched by age, socioeconomic class and area of residence. Control groups individuals were excluded if they had a history of prior antituberculosis therapy, signs and symptoms of suggestive active TB. The diagnostic criteria for TB were defined as the presence of a positive smear for acid fast bacilli [1] and/or culture positivity for *M. tuberculosis* in a sample from sputum and/or bronchial lavage and/or other clinical specimens according to [26]. HIV-infected people and those taking immunosuppressant drugs were excluded from participation in the study. The protocol was approved by the Research Ethics Committees in Brazil (IPEC REC ref. 0008.0.009.000-04) and Rio de Janeiro Municipal Health Centre (REC ref. S/CRH/DRH/DIC3). Ethnic background was determined for each case and control volunteers by self-identification. We recognize the inherent inaccuracy and potential bias in dichotomous self-assessment of ethnic origin, in an admixed population, but self-assessment might nevertheless lead to statistically significant differences between the two groups. All TB patients and control groups were negative for HIV 1/2 infection (following standard diagnosis from The Brazilian Ministry of Health). Tuberculin skin test (TST) response to 5UT RT-23 (Statens Serum Institute, Denmark) was performed, and the skin test response was measured at the diameter of induration 72 h after the injection. Positive results were obtained when induration was ≥10 mm. Control group was classified into those who were naturally infected with *M. tuberculosis *(latency) and those who were uninfected (TST < 10 mm). BCG vaccination status was determined by the presence of the scar tissue. Blood samples were taken after informed consent was obtained from each subject. Patients and controls from the same family were not enrolled in the study.

### 2.2. Genotyping of *IFNG+874T/A* and *NOS2A-954G/C* Gene Polymorphisms

Genomic DNA was extracted from fresh or frozen EDTA blood using a DNA purification kit (QIamp DNA mini Kit, Qiagen, USA) according to the manufacturer's instructions. The *IFNG+874T/A* SNP was detected by amplification refractory mutational system (ARMS-PCR) [9]. The *NOS2A*-*954G/C* SNP was detected by restriction fragment length polymorphism (RFLP) [23]. Amplifications were performed in a 9700 Thermocycler (96-Well GeneAmp PCR System 9700, Applied Biosystems, USA) using 2.5 UI and 1.5 UI for *IFNG *and *NOS2A *of Taq DNA polymerase, respectively (GoTaq flexi DNA polymerase, Promega, USA). Cycling PCR conditions for *IFNG+874T/A* were 3 minutes at 95°C followed by 10 cycles at 95°C for 15 s, 65°C for 50 s, and 72°C for 40 s; 20 cycles at 95°C for 20 s, 55°C for 50 s, and 72°C for 50 s; 72°C for 7 minutes and 4°C until use. Cycling PCR conditions for *NOS2A*-*954G/C* were 3 minutes at 95°C followed by 30 cycles at 94°C for 10 s, 60°C for 30 s, and 72°C for 30 s; 72°C for 7 minutes and 4°C until use. The amplified products were evaluated by electrophoresis on a 1.5% (*IFNG*) and 2.5% (*NOS2A*) agarose gel containing ethidium bromide (0.5 *μ*g/mL).

### 2.3. Detection of Serum Nitric Oxide Radicals Concentration in TB Patients

The two primary stable nonvolatile breakdown products of NO are nitrite (NO_2_^−^) and nitrate (NO_3_^−^). First, the serum NO_2_^−^ levels were determined using a commercial ready to use Griess reaction kit (Promega) according to the manufacturer's instruction. Briefly, serum samples were mixed with sulfanilamide solution following incubation at room temperature. N-1-Naphthylethylenediamine dihydrochloride was added, and absorbance was measured in a plate reader with a filter of 420 nm. Values were plotted in accordance with a NaNO_2_ standard curve (0.8–100 mM). Further analysis was performed to determine the total levels of NO_2_^−^ by reduced NO_3_^−^, in a Vanadium III reduction assay, following a protocol previously described by Miranda et al. [27]. Vanadium III (Sigma, 400 mg in 50 mL of 0.1 N HCl) was added to each well, incubated for 90 minutes at 37°C, and read at 540 nm to determine the total amount of NO_2_^−^ + NO_3_^−^ (after NO_3_^−^ reduction to NO_2_^−^), which was named NO_*x*_^−^. 

### 2.4. Statistical Analysis

Deviation from Hardy-Weinberg equilibrium for the genetic variants was assessed by the chi-square test (*χ* ^2^) in both case and control groups. We used the *χ* ^2^ test to compare the differences in each genotype, allele, and combined genotype of *IFNG+874T/A* SNP and *NOS2A-954G/C* SNP frequency. Additionally, unconditional univariate and multivariate logistic regression analyses were used to examine the associations between the selected SNPs and tuberculosis risk by estimating odds ratios (ORs) and 95% confidence intervals (CIs) with and without adjustment for gender, ethnicity, TST status, and previous BCG vaccination between TB and control groups. All statistical tests were two-sided, a *P* value of ≤0.05 was considered significant, and analyses were performed using Epi Info 6 (Version 6.04, July 1996, CDC, Atlanta, GA, USA), SNPStats (http://bioinfo.iconcologia.net/SNPstats), and SPSS (Version 16, September 2007). Additionally, the distributions of *IFNG+874T/A* and *NOS2A-954G/C* SNPs were compared among patients and in whom TST was positive in the control group by the *χ* ^2^ or Fisher's exact test. Subgroup analyses for genotype, allele, and combined genotype associations to tuberculosis were also conducted among TST-positive individuals. The analysis of the skin test positive group was planned because it was thought that this would represent people with probable latent TB infection. ANOVA test was used to compare the nitrite and NO_*x*_^−^ levels in association with *NOS2A-954G/C *SNP and combined *IFNG+874T/A/NOS2A-954G/C* SNPs genotypes with the level of significance set at *P* < 0.05. 

## 3. Results 

### 3.1. Study Population

TB patients and control group were enrolled consecutively and included 105 males (61.0%) and 67 females (39.0%) with a mean age of 36.9 ± 12.7 years (mean ± standard deviation) in the TB group, and 63 males (35.2%) and 116 females (64.8%) with a mean age of 35.1 ± 11.5 years in the control group. Age was not significantly different between the groups. Each volunteer defined his or her own ethnic group as White (Caucasian) or non-White (Afro-descendants). No Indians or people with Asian background were identified in the included subjects. In the TB group, 136 of 172 (79.1%) individuals defined their ethnic group as White (52, 30.3%) or non-White (84, 48.8%), and among control group, 78 (43.6%) and 82 (45.8%) of 160 were White or non-White, respectively. TST ranged from 0 to 35 (14.9 ± 10.3) mm and from 0 to 57 (12.1 ± 12.2) mm in TB and control groups, respectively. TST-positive (≥10 mm) reaction was identified in 98 of 155 (54.7%) tested subjects from control group, confirming the highest *M. tuberculosis* exposure in Rio de Janeiro. No statistical significance in relation to TST positivity and ethnicity or gender was observed in either TB patients or control groups (Table 1). 

### 3.2. *IFNG+874T/A* SNP Distribution Is Not Associated with Tuberculosis

The genotype distribution of *IFNG+874T/A* SNP was in Hardy-Weinberg equilibrium (*P* > 0.05) in both TB and control groups. TB patients and control group had very similar genotype and allele distributions (*P* > 0.05) (Table 2). No statistical difference in genotypes was observed between TB patients and control subgroup who were TST positive (Table 2). The ability to respond to TST did not correlate with *IFNG+874T/A *SNP genotypes in either TB patients or control group. There is no statistical difference in *IFNG+874T/A *SNP genotypes between TB patients and control groups from univariate or multivariate analysis regarding ethnicity, age, and BCG vaccination status.

### 3.3. *NOS2-954G/C* SNP Distribution Is Not Associated with Tuberculosis

The genotype distributions of *NOS2A-954G/C* SNP were in Hardy-Weinberg equilibrium (*P* > 0.05) in both TB and control groups. No association was seen in the genotypes and alleles frequencies between TB patients and control groups and from those positives TST (control subgroup) (Table 2). When univariate analysis was performed by age, gender, ethnicity, and BCG vaccination, no statistical difference was observed (*P* > 0.05) (Table 2). 

### 3.4. Serum Nitrite and NO_x_^−^ Levels Are Not Associated with *NOS2-954G/C* Polymorphism in Tuberculosis Patients

**Figure 1:**
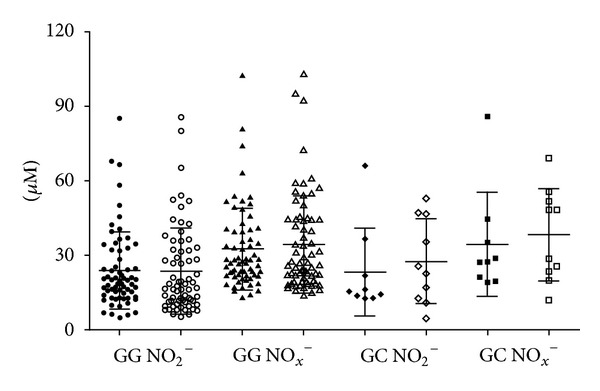
Comparative analysis of serum nitrite (NO_2_^−^) and NO_*x*_^−^ (NO_2_^−^ + NO_3_^−^) concentrations and *NOS2A*-*954G/C* genotype association in tuberculosis patients (black symbols) and control group (open symbols) (*P* > 0.05).

The Griess reaction was performed in 75 TB patients and 78 control group, in proportion to sample sizes of the two groups. The mean serum concentration of nitrite and NO_*x*_^−^ exhibited no statistical difference between TB patients (23.68 ± 15.74 *μ*M and 32.61 ± 16.97 *μ*M) and control group (23.99 ± 17.29 *μ*M and 34.71 ± 19.39 *μ*M), respectively. It was investigated whether serum levels of nitrite and NO_*x*_^−^ could vary according to *NOS2A-954G/C* SNP genotypes in TB patients and control group. No statistical association of serum nitrite (23.77 ± 15.59 versus 23.49 ± 17.39 and 23.13 ± 17.78 versus 27.40 ± 17.08, in TB patients and control group, resp.) or NO_*x*_^−^ levels (32.40 ± 16.6 versus 34.22 ± 19.59 and 34.34 ± 20.99 versus 38.12 ± 18.67, in TB patients and control group, resp.) was observed with *GG* and *GC* genotypes of *NOS2A-954G/C *SNP (Figure 1) even in the TST-positive control subgroup (data not shown) was found. These results suggested that the low *NOS2A-954G/G* SNP or moderate *NOS2A-954G/C *SNP nitric oxide producers were not associated with the modulation of nitrite and NO_*x*_^−^ radical production in either TB patients or control group. The frequency of higher nitric oxide radical producers *NOS2A-954C/C *SNP was rare in our population. 

### 3.5. Combined Genotype of *IFNG+874T/A* and *NOS2A-954C/G* SNPs Is Not Associated with Either Tuberculosis or Nitrogen Radicals

To determine whether the combination of SNPs in these two genes was associated with susceptibility to TB, polymorphisms association was evaluated between pulmonary TB and control groups. The rare *NOS2A-954C/C* SNP was not taken into account in assessing the combined genotypes with the *IFNG+874T/A* SNP. No statistical significance was seen between TB and control groups* in IFNG+874T/A* and *NOS2A-954G/C* SNPs genotypic distributions (Table 3) when they were compared. In both groups, the most prevalent combined genotypes were *AT/GG *(40.7% and 44.7%) and *AA/GG* (36.0% and 31.3%) in TB and control groups, respectively, with no statistical difference. The secretion of reactive nitrogen radicals was not related with the combined genotypes when TB and control groups were compared. The most frequent combined genotype *AT/GG *in TB (23.63 ± 1.60 and 32.35 ± 1.63) and control groups (24.46 ± 1.90 and 34.83 ± 2.21) did not induce different serum levels of nitrite and NO_*x*_^−^. These results demonstrated in our population that the combined genotypes profiles of *IFNG+874T/A* SNP (low *IFNG+874AA*, moderate *IFNG+874AT,* or high *IFNG+874TT *producers) and *NOS2A-954G/C *SNP (low *NOS2A-954GG* or moderate *NOS2A-954GC *producers) are not associated with the modulation of the production of nitrite and NO_*x*_^−^ radicals in both TB patients and control group or in TST-positive control subgroup. 

## 4. Discussion 

IFN-*γ* mediated immune activation has an important role in immunity to intracellular pathogens. IFN-*γ* is critical to macrophage activation, and measurable levels are lower in patients with active TB than in control group [28, 29], but they are not correlated with protection [30]. It has been more formally suggested that IFN-*γ* activity is a continuous, genetically controlled trait with genetic variability in both production and responsiveness to IFN-*γ* [31], although there is little evidence to support a role for variability in IFN-*γ* production. For the *IFNG *gene, there are two intronic SNPs that contribute to its expression phenotypes: *+874T/A *SNP and *+2109G/A* SNP [32]. The *AA* genotype of *IFNG+874T/A *SNP is thought to confer a low-secretor phenotype of IFN-*γ*. Conversely, the *TT* genotype of *IFNG+874T/A *SNP is thought to confer a high-secretor phenotype [33, 34]. Some controversy exists concerning association of *IFNG* gene polymorphisms and susceptibility to pulmonary TB [14, 16, 35–37]. Our results showed no evidence of an association between *IFNG* gene polymorphism and susceptibility to TB. The *AA *genotype frequency of *IFNG+874 *in the control group (35.2%) was a little higher than that in the Sicilian (26%) [32], Spanish (28%) [30], and Indian populations (11%) [38] but was lower than that in South African (47%) [35], Hong Kong Chinese (46%) [36], and South Korean populations (74%) [16]. Reports describing the *T* allele frequency (*IFNG+874T/A *SNP) revealed that there are ethnic differences at this allele distribution. It has been reported that the *T* allele frequency is significantly lower in a Japanese population (9%) [11] than in a South African population (32%) [10] and in an Italian Caucasian population (47%) [39]. Rossouw et al. [35] reported a significantly lower *T* allele frequency* of INFG+874T/A *in patients with *M. tuberculosis* infection than that in control groups in a South African population with a high annual incidence of TB. 

Lio et al. [40] and Vallinoto et al. [41] found that the *TT* genotype was relatively rare in TB patients from Sicilian and Brazilian populations. However, a study conducted in a Central West Brazilian population by Amim et al. [12] showed different results from our study; in that population, the *AA* genotype of* IFNG+874T/A *SNP was associated with TB susceptibility. Amim et al. [12] compared two different populations in their study: one from Rio de Janeiro (patients) and the other from Central West of Region Brazil (control group), which can be characterized as bias because Brazil was colonized by several ethnic groups that migrated from different countries into different regions of Brazil. During the last 500 years these populations became mixed with native Indians, and each Brazilian region has its own admixes ethnic group descendants. Our patients and control group were enrolled from Rio de Janeiro City, which is composed of migratory populations from all over the country. Thus, the population in this city has a genetic background that represents a mix of all regions of the country. 

There is convincing evidence that NO and related reactive nitrogen intermediate (RNI) can kill and/or inhibit intracellular pathogens such as mycobacteria. IFN-*γ* knockout mice which are not capable of producing NO and RNI in response to the bacilli develop tuberculosis quickly, suggesting a role for NO and RNI in the defense mechanism against *M. tuberculosis* [42]. In contrast to murine models of TB, there is greater controversy about the role of NO in killing or limiting the growth of *M. tuberculosis *in humans. Nevertheless, alveolar macrophages from TB patients express higher amounts of NOS2 compared to control group [22], demonstrating a possible role in affecting bacilli growth. Several SNPs have been described in this gene [24] given the importance of this gene in the immune response to TB. Our study verified the influence of *NOS2A-954G/C *SNP on the risk of developing TB in a Brazilian population from a highly endemic area. We did not observe a statistical difference between the *NOS2A-954G/C *SNP genotype in our TB patients and control group. The frequency of *NOS2A-954G/C *SNP may differ among populations. The *C* allele of *NOS2A-954G/C * SNP has been shown to be absent from Caucasian populations [43] and in Peruvian population [44] and in low frequency in Asia [23]. However, the *C* allele has high frequency in African populations [23]. A single description of *NOS2A-954G/C *SNP genotype study in Brazilian population, studying gastric cancer, showed different frequencies for* GG*, *GC,* and *CC* genotypes in *NOS2A-954G/C *SNP (64.77%, 28.69%, and 6.54%, resp.) [45] compared with our study. In Mexicans, this SNP was not associated with TB and *C* allele frequency was 5% [25]. 

The involvement of *NOS2A* SNPs has been studied in different pathologies and still has controversy results. In order to assess the nitric oxide radical levels and to compare them with *NOS2A-954G/C* SNP and combined genotypes of *IFNG+874T/A* SNP*/NOS2A-954G/C* SNP in TB patients and control groups, the secretions of nitrite and NO_*x*_^−^ radicals were analyzed. No association was identified between TB and control groups or between different genotype profiles or combined genotypes regarding nitrite and NO_*x*_^−^ production. Our results indicate that in our admixed population the association between disease development and *IFNG+874T/A* and *NOS2A-954G/C* SNPs does not exert selective pressure on *M. tuberculosis* via immune response surveillance, as shown by the absence of correlation of active TB with these SNPs profiles but can lead to different approaches to evaluate the tuberculosis immunopathology genetics background in the near future. Tuberculosis is a multifactorial disease and the pulmonary milieu involving the immune response and bacilli interaction in different genetic background and environment factors should be addressed to answer the question whether *M. tuberculosis* growth is controlled by other interaction candidate genes and/or several risk factors. 

## Figures and Tables

**Table 1:** Clinical data from Brazilian tuberculosis patients and control group.

	Tuberculosis patients *n* = 172 (%)	Control group *n* = 179 (%)
Sex		
M	105 (61.0)	63 (35.2)
F	67 (39.0)	116 (64.8)
Age (years)	36.9 ± 12.7	35.1 ± 11.5
Skin color		
White	52 (30.3)	78 (43.6)
Non-White	84 (48.8)	82 (45.8)
Missing	36 (20.9)	19 (10.6)
BCG vaccine		
Yes	104 (60.5)	149 (83.2)
No	16 (9.3)	6 (3.3)
Missing	52 (30.2)	24 (13.5)
TST		
Positive	60 (34.9)	98 (54.7)
Negative	24 (13.9)	57 (31.8)
Missing	88 (51.2)	24 (13.5)
Diameter (mm)	(14.9 ± 10.3)	(12.1 ± 12.2)

TST: tuberculin skin test.

**Table 2:** Distribution of genotypes and alleles frequencies of the *IFNG+874T/A* and *NOS2A*-*954G/C* SNPs in tuberculosis patients and control groups.

Genotype/allele	Tuberculosis patients *n* = 172 (%)	Control group *n* = 179 (%)	*P* ^1^	OR	TB latent infection (TST+) *n* = 98 (%)	*P* ^2^	OR
*A/A *	72 (41.8)	62 (34.6)	0.21	1.00	38 (38.8)	0.76	1.00
*A/T *	78 (45.3)	91 (50.8)		0.74	48 (49.0)		0.86
*T/T *	22 (12.8)	26 (14.6)		0.73	12 (12.2)		0.77
*T/T+A/T *versus *A/A *	100 (58.1)	117 (65.3)	0.16	0.74 (0.47–1.16)	60 (61.2)	0.61	0.88 (0.51–1.51)
*T/T *versus *A/A+A/T *	150 (87.2)	153 (85.4)	0.63	0.86 (0.45–1.66)	86 (87.8)	0.89	1.05 (0.47–2.38)
Alleles							
* A *	222 (64.5)	215 (60.1)	0.22	1.21 (0.88–1.66)	124 (63.3)	0.76	1.06 (0.72–1.55)
* T *	122 (35.5)	143 (39.9)			72 (36.7)		
*G/G *	152 (88.4)	160 (89.4)	0.77	1.00	84 (85.7)	0.78	1.00
*G/C *	19 (11.0)	18 (10.0)		1.11	13 (14.3)		0.81
*C/C *	1 (0.6)	1 (0.6)		1.05	0		NA
*C/C* versus *G/C+G/G *	171 (99.4)	178 (99.4)	0.97	1.04 (0.0–38.36)	97 (100)	0.45	—
*G/G *versus *C/C+G/C *	20 (11.6)	19 (10.6)	0.76	0.90 (0.44–1.84)	13 (13.3)	0.67	1.18 (0.52–2.63)
Alleles							
* G *	323 (93.9)	338 (94.4)	0.76	0.91	183 (93.4)	0.80	1.09
* C *	21 (6.1)	20 (5.6)		(0.46–1.66)	13 (6.6)		(0.50–2.35)

*P* value considered *P* ≤ 0.05; OR, odds ratio

*P* ^1^ value from TB patients and control group. *P* ^2^ value from TB patients and control TST-positive subgroup. TST+: tuberculin skin test positive.

**Table 3:** Combined genotype analysis of *IFNG+874T/A* and *NOS2A-954G/C *SNPs in tuberculosis patients and control groups.

Combined genotypes	Tuberculosis patients *n* = 172 (%)	Control groups					
		*n* = 179 (%)	*P* ^1^	OR	TST+ *n* = 98 (%)	*P* ^2^	OR
*AA/GG *	62 (36.0)	56 (31.3)	0.40	1.00	33 (33.7)	0.79	1.00
*AA/GC *	9 (5.2)	5 (2.8)		1.63	4 (4.1)		1.20
*AA/CC *	1 (0.6)	1 (0.6)		0.90	1 (1.0)		0.53
*AT/GG *	70 (40.7)	80 (44.7)		0.79	39 (39.8)		0.96
*AT/GC *	8 (4.7)	11 (6.1)		0.66	9 (9.2)		0.47
*TT/GG *	20 (11.6)	24 (13.4)		0.75	12 (12.2)		0.89
*TT/GC *	2 (1.2)	2 (1.1)		0.90	0 (0)		ND

The *AA/GG* combined genotype was used as reference in a 7 × 2 *χ* ^2^ for trend table. The combined genotypes *TT/GG* and *AT/GG* were not present.

*P-*value considered *P* ≤ 0.05; *P* ^1^-value comparing TB patients and control group.

*P* ^2^ *-*value comparing TB patients and TST+ control subgroup.

TST+: tuberculin skin test positive; OR: odds ratio; ND: not determined.
